# Steps toward the implementation of neurofilaments in multiple sclerosis: patient profiles to be prioritized in clinical practice

**DOI:** 10.3389/fneur.2025.1571605

**Published:** 2025-03-28

**Authors:** Diego Centonze, Alessia Di Sapio, Vincenzo Brescia Morra, Elena Colombo, Matilde Inglese, Damiano Paolicelli, Marco Salvetti, Roberto Furlan

**Affiliations:** ^1^Department of Systems Medicine, Tor Vergata University, Rome, Italy; ^2^IRCCS Neuromed, Pozzilli, Italy; ^3^Department of Neurology, Multiple Sclerosis Regional Referral Centre (CReSM), University Hospital San Luigi Gonzaga, Orbassano, Italy; ^4^Multiple Sclerosis Clinical Care and Research Centre, Department of Neurosciences, Reproductive Sciences and Odontostomatology, Federico II University, Naples, Italy; ^5^Multiple Sclerosis Centre, IRCCS Mondino Foundation, Pavia, Italy; ^6^Department of Neuroscience, Rehabilitation, Ophthalmology, Genetics, and Mother-Child Health (DINOGMI), University of Genoa, Genoa, Italy; ^7^IRCCS Ospedale Policlinico San Martino, Genoa, Italy; ^8^Department of Translational Biomedicines and Neurosciences, University of Bari Aldo Moro, Bari, Italy; ^9^Department of Neurosciences, Mental Health and Sensory Organs, Sapienza University of Rome, Rome, Italy; ^10^Vita e Salute San Raffaele University, Milan, Italy; ^11^Clinical Neuroimmunology Unit, Division of Neuroscience, Institute of Experimental Neurology, IRCCS San Raffaele Scientific Institute, Milan, Italy

**Keywords:** neurofilament light chain, blood NfL, multiple sclerosis, MS biomarker, clinical practice

## Abstract

Multiple sclerosis (MS) is a chronic central nervous system disease characterized by neurodegeneration and inflammation. Neurofilament light chain (NfL), a protein released during axonal injury, has gained recognition as a potential biomarker for monitoring MS progression and treatment response. Evidence indicates that blood NfL (bNfL) offers a minimally invasive, cost-effective tool for tracking neuroaxonal damage. Regular bNfL assessments can identify subclinical disease activity, guide treatment intensification, and support individualized care. However, bNfL level evaluation is currently not optimized in Italian clinical practice. This work examines the utility of bNfL monitoring in clinical practice, focusing on optimizing its use within specific patient profiles, especially in resource-limited settings. bNfL testing, particularly in targeted MS patient profiles, including stable patients exhibiting subclinical signs of disease activity, such as fatigue, and patients off-treatment, represents a promising adjunct for personalized disease management. Its integration into clinical practice, alongside MRI and clinical assessments, can enhance decision-making and improve care efficiency, especially in settings with limited MRI resources. Further research is needed to standardize testing protocols and establish disease-specific cutoffs.

## 1 Introduction

Multiple sclerosis (MS) is a chronic inflammatory disease affecting the central nervous system (CNS) (1), characterized by neuroaxonal damage that correlates with clinical events, magnetic resonance imaging (MRI) findings, and disease prognosis (2). Neurofilament light chain (NfL), a structural protein within the neuronal cytoskeleton, mainly localized in axons but also found in neuronal cell body (3), is crucial for maintaining neuronal integrity in both the central and peripheral nervous system. Neuro-axonal injury leads to the release of NfL into cerebrospinal fluid (CSF), reflecting underlying neurodegenerative and inflammatory processes (4). This release is seen in MS (5) as well as in other conditions such as Alzheimer’s disease and amyotrophic lateral sclerosis (4). Elevated NfL levels indicate neuro-axonal damage, regardless of the primary cause (6).

Although most NfL remains within the CSF, approximately 2% is released in the blood (6), allowing blood NfL (bNfL) to be used as a minimally invasive biomarker. Technological advancements, such as single-molecule array (SiMoA) assays (7, 8) and more recent immunometric assays, have enabled the detection of NfL in serum and plasma at picogram/milliliter concentrations (9), facilitating its use in clinical and research settings (4, 5).

Retrospective cohort studies (10–15) and phase 2 and 3 clinical trials in relapsing and progressive MS (16–20) have demonstrated the promise of NfL as a biomarker. High correlations between CSF and bNfL levels (10, 13, 14) suggest that blood-based measurements of NfL can reliably reflect neuroaxonal damage, making bNfL a useful tool for monitoring disease activity (10, 11).

In patients with relapsing and progressive MS, elevated bNfL concentrations have been observed compared to healthy controls (10, 11). Levels of bNfL correlate with clinical and MRI indicators of inflammatory disease activity, including baseline T2 lesion volume and number (11, 21, 22), T1-hypointense lesion and volume (23, 24), and the presence and quantity of gadolinium-enhancing (Gd+) lesions (10, 25).

Similarly, pediatric MS patients exhibit elevated bNfL levels during their first clinical demyelinating episode (26), with higher levels correlating with MRI activity, relapses, and EDSS scores, mirroring the correlations seen in adults (27). Additionally, the initiation of effective treatment leads to a reduction in bNfL levels, as demonstrated in both clinical trials and real-world studies (6).

Currently, NfL is primarily used as a secondary outcome measure in clinical trials for MS therapies (28), and further validation is needed before it can be routinely adopted in clinical practice. NfL assays are still designated for research-oriented use in most neurodegenerative diseases.

Despite its promising role in MS management and its potential for widespread clinical adoption, the practical use of bNfL testing remains limited. Consensus documents have outlined potential clinical applications for bNfL analysis in MS, providing recommendations on its role, optimal timing, and factors influencing bNfL levels, such as age and comorbidities (29–31). Several challenges, however, must be addressed before it becomes a routine practice, including the need for standardized assay protocols, consistency in the timing of sample collection, and consideration for confounding factors like age, comorbidities, and BMI (5, 29).

## 2 Methods

Given the potential of bNfL to improve MS management, and the source-limitation of territorial clinical settings in Italy, a panel of Italian experts in the management of MS discussed specific scenarios that should be prioritized for bNfL testing in clinical practice. Experts gathered in two expert meetings that were guided by a moderator. The first meeting, held in Rome in June 2024, discussed the value of bNfL measuring, investigated the characteristics of the MS patient to be prioritized for the bNfL evaluation, and the barrier to bNfL use. During the second virtual meeting, held in July 2024, the experts agreed on the patients’ characteristics emerged in the first meeting. Their goal was to provide practical recommendations to guide decision-making and optimize patient outcomes as this biomarker is widely adopted.

## 3 The prognostic value of bNfL for disease activity and progression in MS

High bNfL levels also have a prognostic value, predicting severe clinical outcomes and MS progression during acute disease activity, including relapses (10, 11, 32), development of Gd+ T1 lesions (17), new T2-weighted lesions (11, 16, 19), increased risk of disability worsening (10, 16, 17, 25), and increased expanded disability status scale (EDSS) scores (11, 25, 32). Additionally, bNfL levels can predict future brain and spinal cord atrophy (6, 11).

bNfL levels are linked to retinal neuroaxonal loss in relapsing–remitting MS (33). Research has highlighted the prognostic value of combining bNfL levels with other metrics, such as optical coherence tomography (OCT)-derived retinal measures (34). Compared to OCT alone, this combination provides enhanced predictive power for disease activity (34). The combination of bNfL, OCT metrics, and clinical scores, such as the Bayesian Risk Estimate for MS at Onset (BREMSO), provides both a positive and negative predictive model for early EDSS progression, demonstrating high specificity and sensitivity (35).

Although bNfL is a well-established and reliable marker of neuroinflammation—a key factor in disability accumulation and disease progression—it may have limitations in accurately reflecting the progression of disability when there is no acute disease activity (36). Nevertheless, some studies (25) have identified associations between plasma NfL levels and disability progression, cognitive decline, and brain volume loss, even in contexts when there is minimal evidence of disease activity. In addition, recent evidence showed that one elevated bNfL dosage in stable patients undergoing therapy for at least 12 months is strongly associated with an increased risk of losing NEDA-3 in the following year (37).

Despite some isolated findings indicating no significant changes in bNfL levels following treatment (38), most of clinical trials and real-world studies have demonstrated that bNfL reflects the efficacy of MS therapies (18–20), reinforcing its value as a biomarker for assessing prognosis and monitoring treatment response.

A growing body of evidence supports the predictive value of bNfL for long-term outcomes in MS (18, 24, 39). Elevated bNfL levels have been associated with future brain atrophy over 2 to 12 years (11, 12, 24, 40, 41), particularly when measured early in the disease course.

Furthermore, bNfL concentrations have been shown to be elevated several years before the clinical onset of MS (42), suggesting its potential for detecting subclinical disease activity and, thus, directly reflecting biological mechanisms of MS. This makes bNfL a more sensitive biomarker for the early detection of disease activity than MRI-based measurements of brain atrophy (18).

Several research groups have investigated the longitudinal role of blood NfL as a marker for ongoing disease activity and treatment response in prospective MS cohorts (43–45). For example, a study by Akgun et al. (44) found that bNfL levels begin to rise approximately 5 months before clinical relapse, peaking at onset and returning to baseline within 4–5 months after remission, underscoring the utility of serial bNfL measurements for proactive disease monitoring. However, while bNfL reliably predicts brain atrophy, its role in forecasting long-term EDSS progression remains debated, with mixed evidence across different patient populations. Elevated bNfL levels have been associated with an increased risk of EDSS worsening over the next 1–3 years (10, 11, 32, 46). Another study (41), with a 10-year follow-up, failed to demonstrate such a correlation despite a clear correlation with brain atrophy. Similarly, the predictive value of bNfL for conversion to secondary progressive MS is still under investigation (39, 47). Studies with long-term follow-ups show a stronger correlation in patients with more aggressive disease (6, 29, 48), where the role of confounders is possibly minimal.

## 4 The emerging role of bNfL as a biomarker in MS: clinical applications and considerations

Although bNfL testing is currently used primarily in research, evidence supports its potential clinical application in MS for disease monitoring and treatment evaluation. As an adjunct tool, bNfL could complement clinical and MRI monitoring in tracking MS activity and informing therapeutic decisions (49). Unlike MRI, which captures structural changes that may lag behind ongoing neuroaxonal damage, bNfL provides more immediate insights into active neurodegeneration, enabling frequent and cost-effective monitoring (31). Additionally, bNfL offers advantages in detecting neuroaxonal injury in regions that MRI may overlook, such as the spinal cord or subtle alterations in brain structure not captured by conventional imaging (31). Notably, elevated bNfL levels remain detectable for approximately 3 months after new lesions develop, whereas Gd+ lesions are typically visible on MRI only for 3–4 weeks. This extended presence of bNfL may provide a longer detection window for disease activity, enhancing monitoring beyond the sensitivity of MRI alone (17).

Integrating bNfL measurements with MRI and clinical assessments offers a more robust, multimodal approach to MS management. This combined strategy enhances the sensitivity and specificity of disease activity detection while supporting more individualized care through well-informed treatment adjustments. A recent study examined the impact of incorporating bNfL in routine practice at a tertiary MS clinic by analyzing clinicians’ decision-making through questionnaire responses (50). The study involved routine bNfL testing in various clinical scenarios, such as monitoring DMTs, assessing new symptoms, and aiding in differential diagnosis (50). Results showed that bNfL measurements influenced clinical decisions in nearly 20% of cases, with the highest impact seen in cases with new symptoms or differential diagnosis concerns (50). Clinicians reported greater decision confidence after reviewing bNfL results, especially when elevated levels were detected, leading to treatment modifications and reduced reliance on additional MRI scans. However, no impact was observed on the estimated efficacy of DMTs (50).

As efforts to develop reference values and disease-specific cutoffs for bNfL (51–53) continue, its role in guiding therapeutic decisions in MS is expected to expand significantly.

There are several advantages of using bNfL as a biomarker, including its stability across different conditions (54), high reproducibility in assays like the SiMoA (7, 8), and lower cost compared to MRI (31).

However, it is critical to recognize and account for factors that influence bNfL levels. For example, bNfL levels show an approximate 2.2% annual increase from age 18 to 70 in healthy individuals, and CSF NfL levels also rise with age (10, 55). Adjusting for these age-related changes using a Z-score calculation may provide a more accurate and sensitive measure of disease progression (30). Additionally, elevated bNfL levels have been associated with low BMI, diabetes, cardiovascular disease, renal impairment, and smoking (30, 56–58). Neurotoxic medications and recent physical trauma or intense physical exertion may also contribute to fluctuations in NfL levels (4). However, these confounding factors can be minimized by tracking bNfL levels longitudinally in the same patient.

Biotin supplements, commonly used by MS patients, were previously considered a potential interference risk for bNfL measurement using SiMoA technology. However, recent studies have shown that the SiMoA assay design effectively mitigates this concern by removing excess biotin during processing, ensuring accurate bNfL measurement (59). A manufacturer’s application note states that biotin concentrations up to 80 μM in serum, plasma, or sample diluent do not impact the performance of SiMoA bead-based assay. Since the highest biotin dose intake of 300 mg/day used in high-dose treatments for MS, corresponds to a maximum serum/plasma concentration of 4.92 μM no significant interference can occur in the assay (60). Moreover, manufacturers of *in vitro* diagnostic tests follow the recognized consensus standards Clinical and Laboratory Standard Institute (CLSI EP07) (61), that provides a structured approach to identifying and verifying interference in bioassays, ensuring that clinical chemistry tests are reliable and accurate.

In summary, while various extrinsic and intrinsic factors may affect bNfL concentrations, careful interpretation and consideration of these factors ensure its reliable use as a biomarker. As consensus guidelines continue to evolve, they provide clearer criteria for interpretation of bNfL levels in the context of disease monitoring and treatment response in MS (29–31).

Currently, several limitations restrict bNfL’s widespread clinical use, including cost consideration, resource allocation, and logistical feasibility. For these reasons, it is essential to establish precise guidelines to determine which patient groups and timeframes are best suited for bNfL analysis (29–31). Although an emerging consensus supports its utility across all MS patients, its use in a limited resource setting should be prioritized in cases where bNfL measurement offers the most clinically actionable insights.

## 5 Discussion/perspective

### 5.1 High-priority MS patients for bNfL monitoring

In clinically stable patients who exhibit subclinical signs of disease activity, such as fatigue, sudden onset of anxiety and depression (62), or early cognitive changes without corresponding MRI findings, bNfL measurements every 4–5 months after 1 year of treatment may serve as an early indicator of disease reactivation (Table 1). This approach could help detect subtle disease progression, prompting more frequent MRI evaluations or a change in therapy. Very high bNfL levels may indicate a need for treatment escalation or closer monitoring, whereas low or normal bNfL levels would support the continuation of the current therapy (30). However, elevated bNfL levels in seemingly stable MS patients should also prompt evaluation of other potential causes, such as head trauma or comorbid conditions like polyneuropathy or cerebrovascular events (4).

**Table 1:** High-priority categories of MS patients for bNfL monitoring in clinical practice settings.

Type of patient	Alert/parameter	Timing	Clinical importance	Actions to consider
On therapy, without typical signs of disease activity	Fatigue, sudden onset of mood disorders (anxiety and depression), signals of initial cognitive impairment with no evident indication in MRI	After 1 year of treatment, every 4/5 months	Evaluation of subclinical disease activity	Advanced/more frequent MRI analysis Change of therapy
Off-therapy (including patients previously treated with CLAD or ALEM)	–	Every 6 months	Monitoring of disease activity	Resumption of therapy or switch to a different treatment depending on the evidence and characteristics of disease activity booster of CLAD/ALEM if previously well-tolerated with good long-term response or in the short term, switch to a different HET
Off-therapy Pregnant Women seeking pregnancy	–	Every 6 months	Monitoring disease activity	Close monitoring/Resumption of pregnancy-safe therapy if necessary
Safety monitoring in JCV+ patients treated with NTZ	Subacute Memory loss and behavioral disturbance, cognitive dysfunction, new focal neurological signs, new-onset seizures	Every 6 months, in the absence of symptoms In the presence of warning symptoms, to complement a negative MRI result	Ameliorating follow-up in patients at risk of PML	NTZ withdrawal

ALEM, alemtuzumab; CLAD, cladribine; HET, High-Efficacy Therapy; MRI, magnetic-resonance imaging; JCV, John Cunningham virus; PML, progressive multifocal leukoencephalopathy; NTZ, natalizumab.

The value of bNfL monitoring is especially pronounced when MRI access is limited, as bNfL levels can help optimize the timing of MRI follow-ups. In these cases, bNfL levels serve as a cost-effective adjunct, potentially minimizing unnecessary MRI scans while offering comprehensive disease surveillance.

For patients in off-treatment phases or those following immune reconstitution therapies such as cladribine or alemtuzumab, biannual bNfL monitoring is particularly relevant (Table 1). Real-world data show that cladribine sustains reduced bNfL levels, confirming its role in limiting axonal damage (20, 63, 64) and validating bNfL as a therapeutic response biomarker. Notably, a study by Manni et al. (64) found an inverse correlation between 12-month bNfL levels and the time to NEDA-3 loss, further supporting bNfL as a predictor of MS progression following cladribine treatment. Similarly, alemtuzumab significantly reduces bNfL levels post-treatment, with effects lasting up to 7 years (65). A pilot study further highlighted that bNfL level fluctuations in alemtuzumab-treated patients corresponded with relapse events or MRI lesion activity, suggesting that long-term bNfL monitoring could help assess the efficacy of immune reconstitution therapies (44). In these cases, stable bNfL levels may signal adequate disease control, whereas elevated levels could trigger MRI evaluations or the resumption of treatment. Regular bNfL monitoring in such patients could also reduce the need for frequent MRI scans, potentially lowering healthcare costs without compromising disease surveillance.

Approximately 15–30% of MS patients may experience relapses during pregnancy, and pregnancy can influence the course of MS, as reviewed in Villaverde-Gonzalez et al. (66). Relapse risk in MS decreases during pregnancy but increases significantly postpartum, especially within the first 3 months of postpartum (66). DMT discontinuation before pregnancy and extended washout periods are factors linked to an increased risk of relapse during pregnancy (66, 67). Although evidence on bNfL dynamics in MS patients during pregnancy is limited, it is known that bNfL levels naturally increase during pregnancy in healthy women. These levels also show a positive correlation with maternal age and tend to be higher in cases of preeclampsia (68). A study showed that in stable patients, bNfL levels in the third trimester were similar to those of healthy pregnant women, however, these levels increased during relapses (69). For women planning pregnancy or currently pregnant and off treatment, bNfL monitoring could provide early warning signs, facilitating proactive disease management with pregnancy-safe DMTs, if deemed necessary (Table 1).

For MS patients treated with natalizumab who test positive for John Cunningham virus (JCV) antibodies, bNfL monitoring every 6 months between MRI scans is advisable to improve follow-up for patients at risk of progressive multifocal leukoencephalopathy (PML) (70) (Table 1). Early detection of PML is associated with improved patient outcomes (71), and bNfL has been proposed as a reliable biomarker for its early identification in MS patients (72).

By focusing on these high-priority patient groups, bNfL analysis can be strategically implemented in a targeted manner to maximize its clinical impact while working within the constraints of current healthcare resources.

### 5.2 Secondary priority MS patients for bNfL monitoring

In an ideal setting with ample resources, more patient groups would benefit from bNfL monitoring. For newly diagnosed or treatment-naïve MS patients, an initial bNfL assessment should be conducted before starting therapy, followed by a re-baseline measurement after therapy initiation. This approach is essential for more accurate longitudinal comparisons in the future, providing a clearer view of disease progression than single-point measurements. Determining the optimal timing for the initial bNfL assessment in treatment-naïve patients, whether during an acute phase or after stabilizing from an acute event, is paramount.

A baseline measurement during a stable phase may provide a more accurate reference for evaluating the effects of subsequent DMTs. If bNfL levels are elevated at baseline, this information could influence the therapeutic approach, potentially supporting an early decision to initiate a more aggressive treatment. Ideally, bNfL levels should be reassessed 6–12 months after starting treatment to evaluate the response, monitor disease progression and guide any necessary therapy adjustments. Due to the significant inter-individual variability in bNfL levels, longitudinal monitoring within individual patients is the most effective approach. In patients starting first-line DMTs, bNfL levels generally decrease within 3–6 months, indicating a reduction in neuroinflammation. Conversely, a sustained elevation of bNfL levels despite treatment may indicate unresolved neuroinflammation and suboptimal therapeutic effects, potentially necessitating treatment adjustments. In such cases, regular monitoring every 3–6 months may provide important insights into the effectiveness of current treatment in controlling disease activity.

In cases of potential relapse mimics, where patients present with symptoms similar to a relapse but lack definitive MRI or objective evidence, bNfL testing can help distinguish between actual inflammatory activity and mimicking conditions.

When minimal evidence of disease activity is observed, either radiological (a mildly positive MRI in a prognostically non-unfavorable location) or clinical (without disability accrual), after a period of long-term disease stability, a closer follow-up is suggested. In these instances, the persistence of elevated bNfL levels may indicate the need for a therapy switch.

Indeed, bNfL levels are reported to gradually decrease following an acute relapse. However, persistently elevated bNfL levels with no noticeable decrease 3–4 months after an acute event may predict a poorer prognosis or an underlying smoldering disease, suggesting that a switch to a higher-efficacy therapy could be advisable.

Using bNfL as a biomarker reflecting disease course can help optimize treatment decisions, minimizing the risks of overtreatment and ensuring timely responses to actual disease activity.

### 5.3 Broader implications and future perspectives

Looking ahead, bNfL is anticipated to play an expanding role in managing diverse neurological disorders beyond MS, offering a valuable biomarker for monitoring the neurodegeneration of different etiologies. Developing biobanks to store bNfL samples could greatly enhance large-scale longitudinal studies, improving our understanding of disease mechanisms and reinforcing the biomarker’s clinical applications. However, successfully integrating bNfL into clinical practice across multiple centers will require the standardization of testing methods and platforms to ensure uniformity and reliability in results.

While bNfL provides key insights into disease activity, it is not yet a standalone diagnostic tool and cannot replace MRI for MS diagnosis or ongoing disease monitoring. Instead, bNfL should be incorporated into a multimodal approach that combines clinical evaluations and imaging findings. Future research should aim to establish disease-specific thresholds, explore the kinetics of bNfL release, and investigate potential combinations with other biomarkers to enhance its prognostic capabilities. Additionally, as highlighted in Table 1, bNfL monitoring holds promise for the early detection of PML, representing another potential application of this biomarker.

## 6 Limitations

Despite its utility, bNfL lacks specificity to MS, as its levels may also rise due to various other neurological conditions or injuries (4), complicating its application in distinguishing MS activity or treatment response. Additionally, specific comorbidities associated with progressive MS may lead to elevated bNfL levels. This lack of specificity may limit bNfL’s ability to accurately capture the neurodegenerative components of progressive MS or reflect the effects of neuroprotective therapies. Possible limitations due to bNfL sensitivity compared to MRI should be considered, as the temporal association with new gadolinium-enhancing lesions needs to be further investigated (73). Understanding the dynamics of bNfLs in MS patients in different conditions will be crucial for advancing its use in personalized MS care. While bNfL is primarily useful for monitoring disease activity in relapsing MS, its applicability in progressive MS remains limited. Emerging evidence suggests that glial fibrillary acidic protein (GFAP) may serve as a complementary biomarker, as it reflects neurodegenerative processes more characteristic of progressive MS (29).

## 7 Conclusion

bNfL levels serve as a powerful biomarker, offering essential insights into the neurodegenerative processes associated with MS. Their stability and capacity to reflect disease activity in real-time hold potential for enhancing personalized care in MS. However, to maximize its clinical impact in resource-limited clinical settings, it is essential to prioritize patient profiles that would benefit the most from bNfL monitoring. A targeted approach to bNfL testing can optimize its benefits, reduce unnecessary interventions, and support a more personalized, cost-effective strategy for MS care.

## Data Availability

The original contributions presented in the study are included in the article/supplementary material, further inquiries can be directed to the corresponding author.
